# Knockdown of TC-1 enhances radiosensitivity of non-small cell lung cancer via the Wnt/β-catenin pathway

**DOI:** 10.1242/bio.017608

**Published:** 2016-03-30

**Authors:** Dapeng Wu, Lei Li, Wei Yan

**Affiliations:** 1 Department of Radiotherapy, Huaihe Hospital of Henan University, Kaifeng 475000, China; 2 Department of Respiratory, Huaihe Hospital of Henan University, Kaifeng 475000, China

**Keywords:** TC-1, Radiosensitivity, NSCLC, Wnt/β-catenin

## Abstract

Thyroid cancer 1 (TC-1, C8ofr4) is widely expressed in vertebrates and associated with many kinds of tumors. Previous studies indicated that TC-1 functions as a positive regulator in the Wnt/β-catenin signaling pathway in non-small cell lung cancer (NSCLC). However, its exact role and regulation mechanism in radiosensitivity of NSCLC are still unclear. The expression level of TC-1 was measured by qRT-PCR and western blot in NSCLC cell lines. Proliferation and apoptosis of NSCLC cells in response to TC-1 knockdown or/and radiation were determined by MTT assay and flow cytometry, respectively. The activation of the Wnt/β-catenin signaling pathway was further examined by western blot *in vitro* and *in vivo*. Compared to TC-1 siRNA or radiotherapy alone, TC-1 silencing combined with radiation inhibited cell proliferation and induced apoptosis in NSCLC cell lines by inactivating of the Wnt/β-catenin signaling pathway. Furthermore, inhibition of the Wnt/β-catenin signaling pathway by XAV939, a Wnt/β-catenin signaling inhibitor, contributed to proliferation inhibition and apoptosis induction in NSCLC A549 cells. Combinative treatment of A549 xenografts with TC-1 siRNA and radiation caused significant tumor regression and inactivation of the Wnt/β-catenin signaling pathway relative to TC-1 siRNA or radiotherapy alone. The results from *in vitro* and *in vivo* studies indicated that TC-1 silencing sensitized NSCLC cell lines to radiotherapy through the Wnt/β-catenin signaling pathway.

## Abstract (precis)

**Summary:** TC-1 silencing inhibited cell proliferation and induced apoptosis in non-small cell lung cancer (NSCLC) both *in vitro* and *in vivo* through the Wnt/β-catenin signaling pathway, thereby increasing the susceptibility of NSCLC to radiotherapy.

## INTRODUCTION

Lung cancer is one of the most prevalent malignancies and the leading cause of cancer-related death worldwide (George et al., 2015; Pérol et al., 2016). Around 90% of patients with lung cancer experienced external-beam radiation therapy (RT), which is currently the potentially curative nonsurgical approach for the treatment of most solid tumors (Henson et al., 2013; Liao et al., 2015). However, therapeutic resistance is the main factor that leads to the treatment failures of cancers, including non-small cell lung cancer (NSCLC) (Aktipis et al., 2011; Cao et al., 2004). Most patients with NSCLC present cellular desensitization, which limits the effectiveness of tumoricidal radiation and in turn results in resistance and recurrence (Brognard et al., 2001; Li et al., 2015). Therefore, finding molecular target or agents to overcome radioresistance may contribute to effectiveness of radiotherapy and, thereby, overall survival of NSCLC.

Thyroid cancer 1 (TC-1, C8ofr4), a novel 106-residue protein, was originally found to be overexpressed in papillary thyroid cancer (Chua et al., 2000; Sunde et al., 2004). TC-1 overexpression in normal thyroid cells increased proliferation rates, enhanced anchorage-independent growth in soft agar, and decreased apoptosis rates (Sunde et al., 2004). Accumulated evidence have shown that TC-1 overexpression was detected in a wide range of solid tumors, including gastric cancer (Kim et al., 2006a), colon cancer (Friedman et al., 2004), breast cancer (Yang et al., 2007), and lung cancer (Su et al., 2013). The data from these studies were involved in the poor cell differentiation and aggressive biological behavior of malignancies. In breast cancer cell lines and tissues, the expression of TC-1 was found to be significantly upregulated, thereby implicating the important role of this protein in the breast cancer development (Ray et al., 2004). TC-1 was also detected to be overexpressed in both gastric cancer cell lines and tissues, and it is correlated with tumor stage, poor differentiation, lymphatic infiltration, lymph node metastasis, and poor survival (Kim et al., 2006a).

Additional studies have indicated that TC-1 is a positive regulator of the Wnt/β-catenin signaling pathway, which are implicated in invasiveness and aggressive behavior of cancers (Jung et al., 2006; Karim et al., 2004; Su et al., 2013; Urakami et al., 2006; Yang et al., 2007). TC-1 activated the Wnt/β-catenin signaling pathway by releasing β-catenin from Chibby, which is a conserved nuclear protein that blocks β-catenin-mediated transcriptions (Kim et al., 2006b; Takemaru et al., 2003). In 299 gastric cancers, TC-1 expression was in correlation with a subset of β-catenin target genes, including laminin γ2, metalloproteinase-7 and metalloproteinase-14, cyclin D1, c-Met, and CD44 (Kim et al., 2006a). High expression of TC-1 was common in 109 cases of oral tongue squamous cell carcinomas (OTSCCs) and correlated with the expression of β-catenin and cyclin D1, indicating the carcinogenesis of TC-1 in OTSCCs by enhancing the activity of Wnt/β-catenin signaling pathway (Zhang et al., 2015). TC-1 was also observed in 97 of the 147 primary tumor specimens of NSCLC, and it was correlated with the TNM stage as well as regional lymph node metastasis, which was in line with the results of *in vitro* experiments demonstrating that it promoted the proliferation and invasion of lung cancer cells through Wnt/β-catenin signaling pathway (Su et al., 2013).

In this study, we investigated the role of targeted deletion of TC-1 in radiosensitivity of NSCLC using a series of *in vitro* and *in vivo* studies. We also assessed whether Wnt/β-catenin pathway was involved in this process.

## RESULTS

### TC-1 expression in siRNA-treated cells

**Fig. 1.**
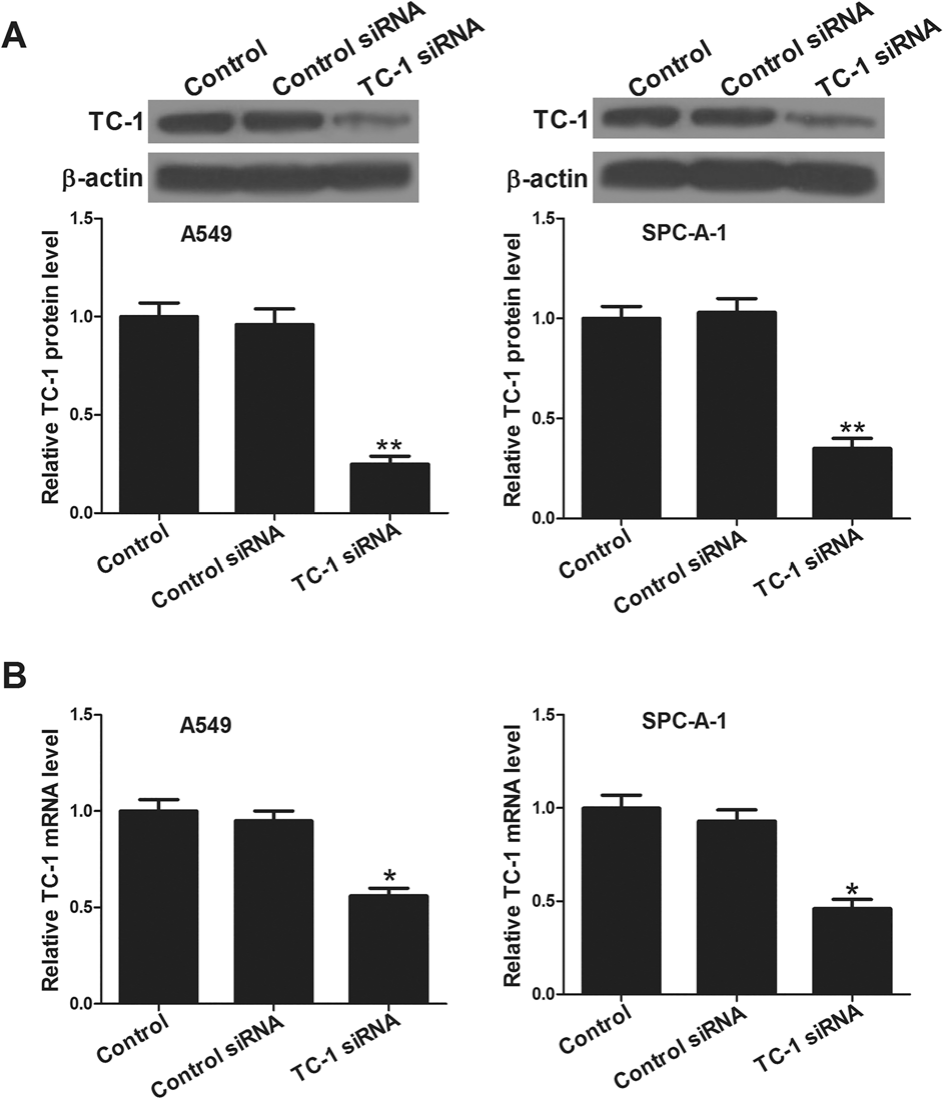
**Detection of TC-1 expression in A549 and SPC-A-1 cells.** (A) After transfection with TC-1 siRNA, the expression level of TC-1 was significantly decreased. (B) qRT-PCR was employed to detect the mRNA levels of TC-1. Mean±s.d.; *P<0.05 and **P<0.01 compared to control group. Control, A549 or SPC-A-1 cells; control siRNA, A549 or SPC-A-1 cells transfected with control siRNA; TC-1 siRNA, A549 or SPC-A-1 cells transfected with TC-1 siRNA; β-actin was used as a loading control.

To study the effect of deletion of TC-1 on proliferation and radiosensitivity, NSCLC cell lines A549 and SPC-A-1 were transfected with TC-1 siRNA or control siRNA, and the stable expression clones were selected. Western blot and qRT-PCR were used to confirm TC-1 expression (Fig. 1A,B). The results showed that the protein and mRNA levels of TC-1 were significantly decreased in cells transfected with TC-1 siRNA.

### Knockdown of TC-1 sensitized NSCLC cell lines to radiation therapy

**Fig. 2.**
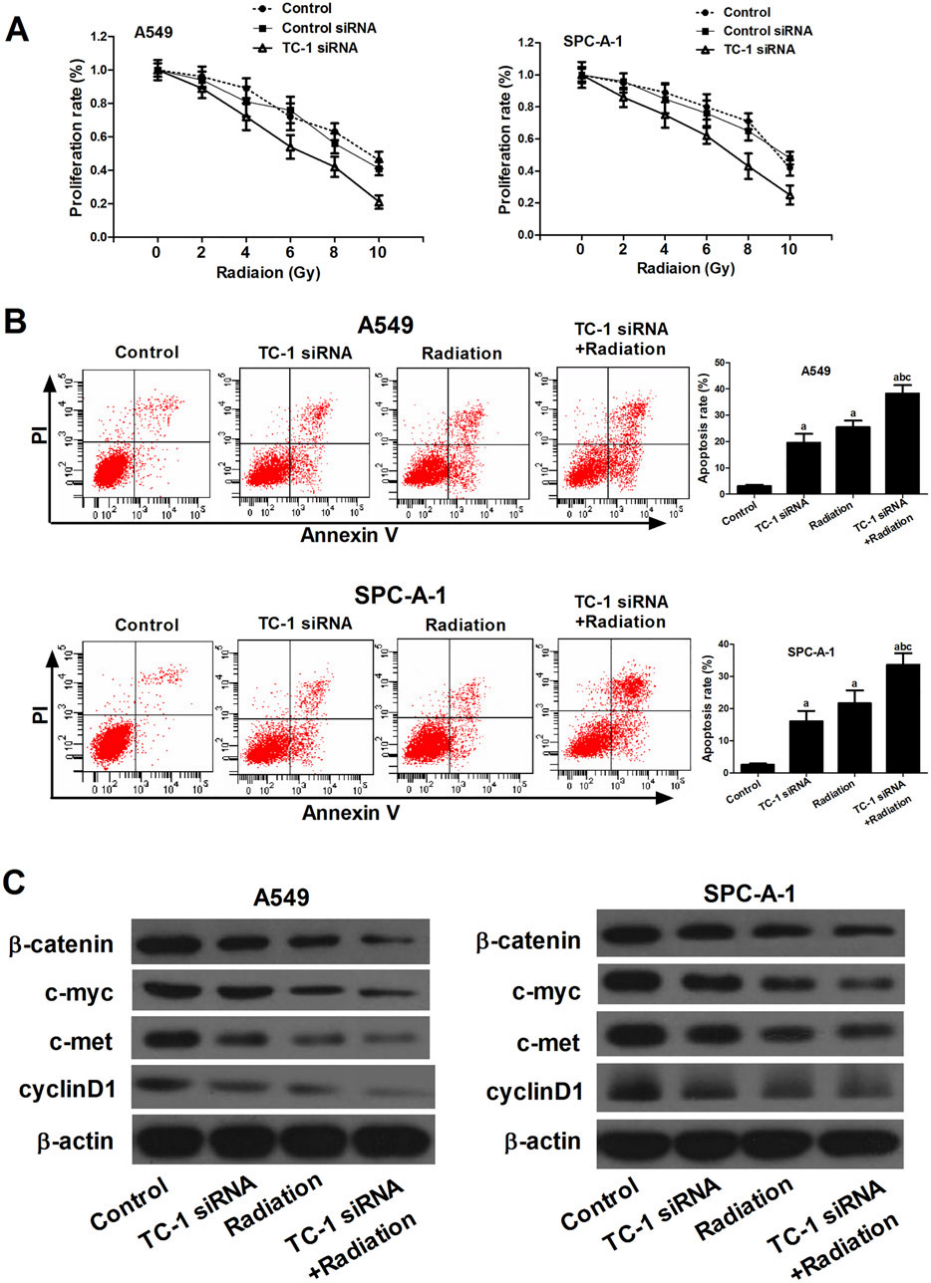
**TC-1 silencing sensitized A549 and SPC-A-1 cells to radiation therapy.** (A) Proliferation rate calculated for A549 and SPC-A-1 cells. (B) Flow cytometry analysis was used to detect the apoptosis in A549 and SPC-A-1 cells treated with siRNA or/and radiation. (C) The expression levels of β-catenin, c-myc, c-met and cyclinD1 in A549 and SPC-A-1 cells treated with siRNA or/and radiation, with β-actin as loading control. Mean±s.d.; ^a^*P*<0.05 compared to control group, ^b^*P*<0.05 compared to TC-1 siRNA group, ^c^*P*<0.05 compared to radiation group.

To investigate the effects of TC-1 silencing on radiation-induced cytotoxicity in NSCLC cell lines A549 and SPC-A-1, MTT assay was used to determine the growth of cells transfected with TC-1 siRNA or control siRNA, and flow cytometry was used to detect the apoptosis of cells treated with TC-1 siRNA alone, radiation alone or TC-1 siRNA combined with radiation. The results showed that all of the two cell lines transfected with TC-1 siRNA displayed lower proliferation rates compared with cells with control siRNA, and control siRNA had no significant effect on the cell proliferation compared with control group (Fig. 2A). Moreover, the apoptosis rates in A549 and SPC-A-1 cells treated with TC-1 siRNA or 4 Gy of X-rays were significantly increased compared with control cells, and the combination treatment showed a stronger inhibitory effect (Fig. 2B).

To further understand the underlying mechanism of this effect, we assessed the activity of the Wnt/β-catenin pathway and the expression of its target genes. Expression levels of β-catenin, c-myc, c-met, and cyclinD1 were measured by western blot to evaluate the effect of TC-1 knockdown on the Wnt/β-catenin signaling pathways (Fig. 2C). In both A549 and SPC-A-1 cells, TC-1 siRNA or radiation alone inhibited the expression levels of these proteins compared with control cells, and combinative treatment potentiated these effects.

### XAV939 inhibited cell proliferation and induced apoptosis in NSCLC cell lines

**Fig. 3.**
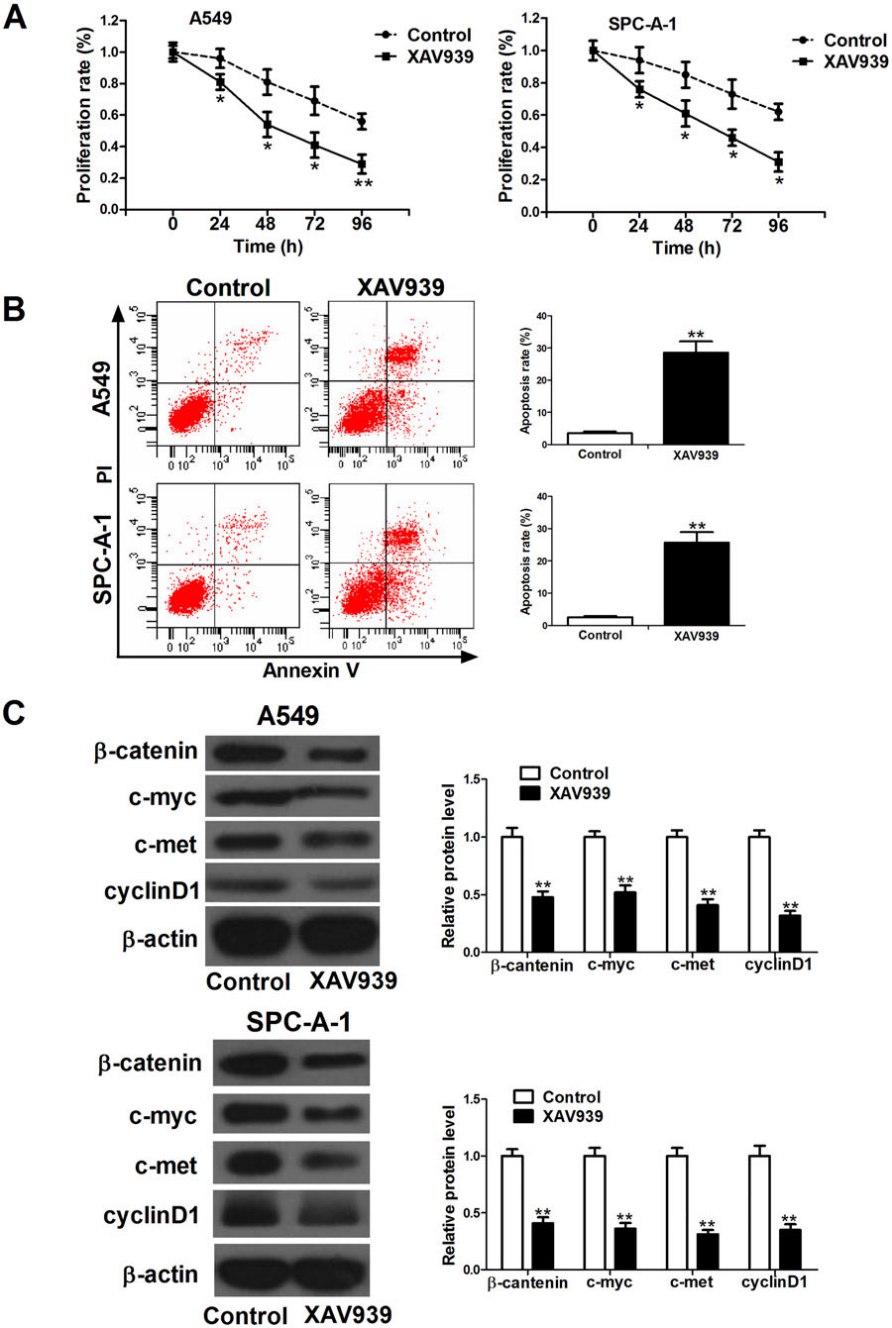
**Inactivation of the Wnt/β-catenin signaling pathway inhibited cell proliferation and induced apoptosis in A549 and SPC-A-1 cells.** Cells were treated with 10 μM Wnt/β-catenin signaling pathway inhibitor, XAV939 for 24 h. (A) XAV939 treatment significantly inhibited the growth of A549 and SPC-A-1 cells compared with control group. (B) XAV939 treatment significantly induced apoptosis in A549 and SPC-A-1 cells compared with control group. (C) The expression levels of β-catenin, c-myc, c-met, and cyclinD1 were markedly down-regulated in XAV939-treated cells compared with control group. β-actin was used as a loading control. Mean±s.d.; *P<0.05 and **P<0.01 compared to control group.

Our data indicated that the Wnt/β-catenin pathway was involved in the effect of TC-1 silencing on radiation-induce anti-tumor therapy. We sought to determine whether the inactivity of the Wnt/β-catenin pathway by XAV939 (a Wnt/β-catenin signaling inhibitor) inhibited cell proliferation and induced apoptosis in A549 and SPC-A-1 cells. The results from MTT assay showed that 10 μM XAV939 (Sigma-Aldrich) significantly inhibited cell proliferation compared with control group after XAV939 treatment for 24, 48, 72, 96 h (Fig. 3A). As shown in Fig. 3B, XAV939 treatment significantly increased apoptosis in NSCLC cells compared with control group (*P*<0.01). Furthermore, the expression levels of β-catenin, c-myc, c-met, and cyclinD1 were markedly down-regulated in XAV939-treated cells compared with control group (Fig. 3C).

### Combination of TC-1 silencing and radiotherapy inhibited tumor growth in A549 tumor xenografts

**Fig. 4.**
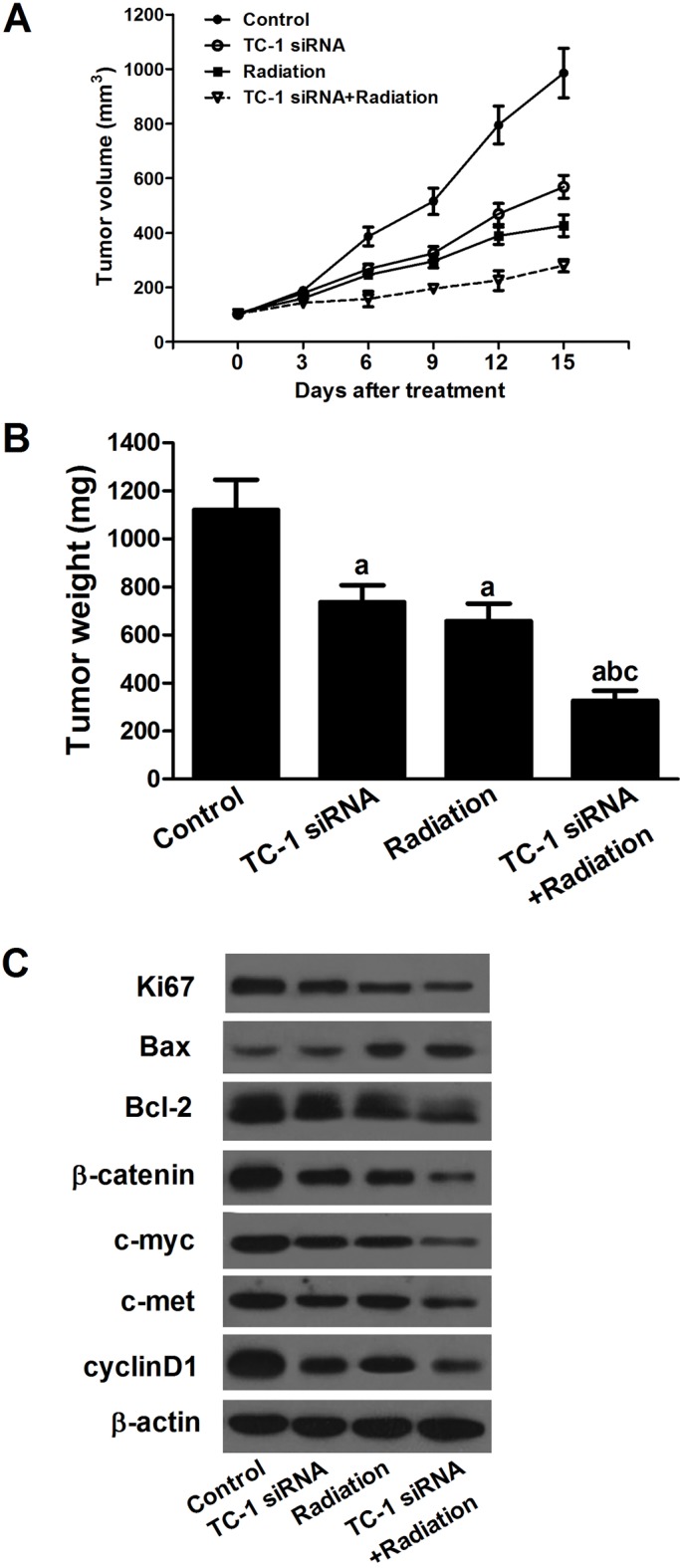
**The effect of combination of TC-1 silencing and radiotherapy on A549 tumor xenografts.** (A) Combination of TC-1 silencing and radiation inhibited the growth of A549 xenografts. (B) Tumor weights were measured when the mice were euthanized 15 days after the treatment. Combination of TC-1 silencing and radiation decreased tumor weight compared with TC-1 siRNA or radiation alone. (C) siRNA or radiation alone significantly increased the Bax expression while decreased the expression levels of Ki67, Bcl-2, β-catenin, c-myc, c-met, and cyclinD1 (with β-actin as loading control), and the effect of combinative treatment was stronger. Mean±s.d.; ^a^*P*<0.05 compared to control group, ^b^*P*<0.05 compared to TC-1 siRNA group, ^c^*P*<0.05 compared to radiation group.

We next examined the effect of TC-1 siRNA alone, radiation alone or TC-1 siRNA combined with radiation on A549 tumor xenografts. TC-1 siRNA alone or radiation alone significantly inhibited the growth of the tumor model compared with control group. However, when TC-1 siRNA combined with radiation, there was a considerable inhibition of tumor growth compared with TC-1 siRNA alone or radiation alone. After the mice were euthanized, tumor weight measurement of A549 tumor xenografts was also carried out, and a similar inhibition in the alone and combinative treatment was observed (Fig. 4B). Moreover, we measured the expression levels of Ki67, Bax, Bcl-2, β-catenin, c-myc, c-met, and cyclinD1 in the A549 xenograft model. The results showed that compared with control group TC-1 siRNA or radiation alone significantly up-regulated the Bax expression while down-regulated the other proteins, and the effect of combinative treatment was stronger (Fig. 4C).

## DISCUSSION

TC-1 has been reported to express in various human tissues like the heart, liver, and lung, which indicated the important role of TC-1 in biological functions (Kim et al., 2009; Wang et al., 2008; Xu et al., 2013; Zhang et al., 2011, 2015). In many kinds of tumors, TC-1 is detected to be overexpressed (Friedman et al., 2004; Kim et al., 2006a; Su et al., 2013; Yang et al., 2007). Data from immunohistochemistry showed that TC-1 was expressed in all ovarian carcinoma samples where high expression of TC-1 was 84%, which correlated with poor differentiation of ovarian carcinoma. Moreover, TC-1 was highly expressed in 100% of nine metastatic ovarian cancer, whereas only 31% of 16 metastatic colorectal cancers was observed (Lee, 2010; Xu et al., 2013). For these studies, we hypothesized that TC-1 knockdown might inhibit aggressive behavior through the Wnt/β-catenin signaling pathway in lung cancer, and thereby sensitizing NSCLC cell lines to radiation therapy. To confirm this hypothesis, in our study, we firstly deleted TC-1 by siRNA transfection in NSCLC cell lines A549 and SPC-A-1. Our data showed that TC-1 silencing combined with radiation inhibited cell proliferation and induced apoptosis in NSCLC cell line relative to TC-1 siRNA alone. The combinative treatment of TC-1 siRNA and radiation showed an even stronger effect on tumor xenografts amelioration in nude mouse models than the TC-1 siRNA alone. Moreover, the Wnt/β-catenin signaling pathway was inhibited both *in vitro* and *in vivo*.

Several studies have shown that TC-1 played crucial roles in cell cycle control and multiple signaling pathways (Jung et al., 2014; Kim et al., 2009; Wang et al., 2008). It has been reported that TC-1 is a positive regulator of the Wnt/β-catenin signaling pathway in many kinds of human cancers, including breast cancer, gastric cancer, and lung cancer. In many cell lines, TC-1 positively regulated β-catenin target genes through interaction with Cby, which is an endogenous inhibitor of the canonical Wnt signaling pathway (Jung et al., 2006; Kim et al., 2006a). At the C-terminus of β-catenin, the Cby binds to the interaction domain of the LEF/TCF transcription complexes and thereby inhibits interaction of β-catenin with LEF/TCF transcription factors, which results in a suppression of β-catenin-mediated transcription within the signaling cascade (Takemaru et al., 2003). TC-1 competes with β-catenin for the interaction with Cby, and thereby up-regulates the signaling pathway through relieving the block by Cby (Yang et al., 2007). Here, after NSCLC A549 and SPC-A-1 cells were treated with siRNA, proliferation rates were significantly decreased and apoptosis rates were increased in cells treated with TC-1 siRNA compared with control group. TC-1 siRNA also inhibited gastric tumorigenesis in nude mouse models. Furthermore, the Wnt/β-catenin signaling pathway was down-regulated by TC-1 siRNA treatment. A far more striking result is that combinative treatment of TC-1 siRNA and radiation showed a stronger inhibitory effect *in vitro* and *in vivo*, indicating the positive role of TC-1 knockdown in potentiating the cytotoxicity of radiation treatment in NSCLC.

A battery of Wnt/β-catenin target genes, including *CLDN1*, *CCND1*, *c-myc*, *c-met* and *cyclinD1*, were highly expressed in cell lines presenting TC-1 over expression (Smalley and Dale, 2001). Our present study also showed that *in vitro* and *in vivo* treatment of NSCLC with TC-1 siRNA caused down regulation of the β-catenin target genes *c-myc*, *c-met* and *cyclinD1.* However, the exact mechanisms regulating Wnt/β-catenin and its target genes are complex and a lot of other factors besides Cby that interact with TC-β-catenin to regulate the transcriptional activation (Clevers, 2006; Gordon and Nusse, 2006). Taken together, our data suggested that targeting pathways that enhance radiosensitivity through down-regulation of TC-1 may represent an effective new strategy for treating lung cancer and perhaps other cancers as well.

In conclusion, the results of the present study suggest TC-1 knockdown inhibited cell proliferation and induced apoptosis in NSCLC *in vitro* and *in vivo* via the Wnt/β-catenin signaling pathway, thereby increasing susceptibility of NSCLC to radiotherapy.

## MATERIALS AND METHODS

### Cell culture

The NSCLC cell lines A549 and SPC-A-1 were purchased from the Cell Bank of Type Culture Collection of Chinese Academy of Sciences (Shanghai, China). The cells were cultured at 37°C in humidified chambers with 5% CO_2_ in RPMI 1640 medium containing 10% fetal bovine serum and penicillin (100 U/ml) and streptomycin (100 µg/ml). Cells were transfected with 100 nmol/l of TC-1 siRNA or control siRNA (Invitrogen, Carlsbad, CA, USA) using Lipofectamine 2000 (Invitrogen) according to the manufacturer's instructions, and stable expression clones were selected by G418 (Sigma-Aldrich, St Louis, MO, USA). Cells were grown under standard conditions for a further 48 h before being examined by qRT-PCR or western blot analysis.

### Radiation treatment

Forty-eight hours after transfection, cells were irradiated with 0, 2, 4, 6, 8, and 10 Gy of X-rays using 6-MeV X-ray linear accelerator (Siemens, Munich, Bavaria, Germany). Cells were cultured for a further 24 h before being examined by MTT assay, flow cytometry and western blot analysis. For tumors, when tumor volume reached the required size (0.8-1.0 cm^3^), mice were immobilized, and tumors were vertically irradiated in the 2.0 cm×2.0 cm radiation field. Radiation was delivered at a dose rate of 4 Gy/min. During therapy, the tumor volume was measured using calipers and calculated as 0.5×largest diameter×smallest diameter^2^ per two days.

### Tumor xenograft and irradiation therapy

Animal experiments were consistent with the regulations of animal use for biomedical experiments issued by Ministry of Science and Technology of China and approved by the Committee on Experimental Animals of Huaihe Hospital of Henan University. Female athymic BALB/c nu/nu mice (6- to 8-week-old, 15-20 g) were purchased from Shanghai Experimental Animal Center (Shanghai, China). Xenografts were generated by subcutaneous injection of 1×10^7^ A549 cells in a volume of 20 µl into the right hind leg of the female athymic BALB/c nu/nu mice. When tumor volume reached the required size, mice were assigned to the following groups (five mice per group): control (no treatment), TC-1 siRNA alone (5 µg siRNA daily for 5 days by intratumoral injection), radiation alone (4 Gy once per day for 5 days, total 20 Gy), or TC-1 siRNA+radiation. TC-1 siRNA was mixed with a PEI (Sigma-Aldrich) according to the manufacturer's instructions was given 1 h before radiation. The final complex was prepared immediately before intratumoral injection. All the mice were euthanized 15 days after the treatment, and the tumors were resected for tumor weight measurement and western blot analysis.

### Quantitative RT-PCR (qRT-PCR)

Total RNA was extracted from cultured cells using Trizol reagent (Invitrogen) according to the manufacturer's protocol. The cDNA was synthesized using the SuperScript First Strand Synthesis Kit (Invitrogen). qRT-PCR analyses were done to measure TC-1 mRNA by using SYBR Green Gene Expression Assay Kit (Qiagen, Valencia, CA, USA). Primer sequences of TC-1 and β-actin were synthesized by Shanghai Sangon Biological Engineering Technology & Services (Shanghai, China). The β-actin mRNA was used as the internal standard and relative quantification was calculated by the 2−ΔΔCt method. Primer sequences used were as follows: TC-1 (c8orf4): forward, 5′-AGCCACCAAGCCATCATCAT-3′; reverse, 5′-TGTGTCGAAGTGGTAGCCATG-3′; β-actin: forward, 5′-TGGCACCCAGCACAATGAA-3′; reverse, 5′-CTAAGTCATAGTCCGCCTAGAAGCA-3′.

### Western blot analysis

Protein concentrations of cell or tumor lysates were measured by the BCA protein assay kit (Thermo Fisher Scientific, USA) and proteins were electrophoresed on SDS-PAGE gels. Separated proteins were then transferred onto PVDF membranes (Millipore, Bedford, MA, USA) followed by the incubation with specific antibodies against β-catenin (BD, Transduction Laboratories, KY, USA), c-myc (Roche Applied Sciences, Indianapolis, IN, USA), c-met (Santa Cruz, Biotechnology Inc., CA, USA), cyclin D1 (Santa Cruz Biotechnology), TC-1 (Santa Cruz Biotechnology), and β-actin (Sigma), and immunoblotted with the appropriate secondary antibody (Cell Signaling, Beverly, MA, USA). Chemiluminescent detection was performed using an ECL system (Amersham Pharmacia, Piscataway, NJ, USA).

### Cell proliferation assay

Cell proliferation was detected using MTT assay. After 48 h of the treatment with TC-1 siRNA, cells (5×10^3^ cells/well) were seeded on 96-well culture plates for 48 h. Then, 20 µl of MTT (5 mg/ml) was added to each well and cells were incubated at 37°C for 4 h. The MTT-supplemented medium was replaced with 150 μl/well of DMSO (Sigma-Aldrich). After shaking the plates for 10 min, the optical density (OD) value was determined at 490 nm. All experiments were repeated three times, and the cell proliferation rates were calculated based on the OD value of non-irradiated cells.

### Flow cytometry analysis of cell apoptosis

Apoptosis of NSCLC cells was detected by the Annexin V-PI Apoptosis Detection Kit (BioVision, California, USA). Briefly, cells were harvested by trypsinization and collected in centrifuge tubes, and washed with ice-cold PBS. Then, the cells were suspended in 500 ml of binding buffer and incubated with Annexin V-FITC and propidium iodide (PI). The apoptosis rate was analyzed by flow cytometry (FACSCalibur; BD, Franklin Lakes, NJ, USA).

### Statistical analysis

All values were expressed as mean±standard deviation (s.d.). One-way ANOVA or Student's *t*-test was applied to evaluated the significant differences. GraphPad Prism 6 software (GraphPad Prism Software, Inc., San Diego, CA, USA) was used for the statistical analysis. *P*<0.05 was considered statistically significant.

## References

[BIO017608C1] Aktipis C. A., Kwan V. S. Y., Johnson K. A., Neuberg S. L. and Maley C. C. (2011). Overlooking evolution: a systematic analysis of cancer relapse and therapeutic resistance research. *PLoS ONE* 6, e26100 10.1371/journal.pone.0026100 22125594 PMC3219640

[BIO017608C2] Brognard J., Clark A. S., Ni Y. and Dennis P. A. (2001). Akt/protein kinase B is constitutively active in non-small cell lung cancer cells and promotes cellular survival and resistance to chemotherapy and radiation. *Cancer Res.* 61, 3986-3997. 11358816

[BIO017608C3] Cao C., Mu Y., Hallahan D. E. and Lu B. (2004). XIAP and survivin as therapeutic targets for radiation sensitization in preclinical models of lung cancer. *Oncogene* 23, 7047-7052. 10.1038/sj.onc.1207929 15258565

[BIO017608C4] Chua E. L., Young L., Wu W. M., Turtle J. R. and Dong Q. (2000). Cloning of TC-1(C8orf4), a novel gene found to be overexpressed in thyroid cancer. *Genomics* 69, 342-347. 10.1006/geno.2000.6348 11056052

[BIO017608C5] Clevers H. (2006). Wnt/β-catenin signaling in development and disease. *Cell* 127, 469-480. 10.1016/j.cell.2006.10.018 17081971

[BIO017608C6] Friedman J. B., Brunschwig E. B., Platzer P., Wilson K. and Markowitz S. D. (2004). C8orf4 is a transforming growth factor B induced transcript downregulated in metastatic colon cancer. *Int. J. Cancer* 111, 72-75. 10.1002/ijc.20235 15185345

[BIO017608C7] George J., Lim J. S., Jang S. J., Cun Y., Ozretić L., Kong G., Leenders F., Lu X., Fernández-Cuesta L. and Bosco G. (2015). Comprehensive genomic profiles of small cell lung cancer. *Nature* 524, 47-53. 10.1038/nature14664 26168399 PMC4861069

[BIO017608C8] Gordon M. D. and Nusse R. (2006). Wnt signaling: multiple pathways, multiple receptors, and multiple transcription factors. *J. Biol. Chem.* 281, 22429-22433. 10.1074/jbc.R600015200 16793760

[BIO017608C9] Henson K. E., McGale P., Taylor C. and Darby S. C. (2013). Radiation-related mortality from heart disease and lung cancer more than 20 years after radiotherapy for breast cancer. *Br. J. Cancer* 108, 179-182. 10.1038/bjc.2012.575 23257897 PMC3553540

[BIO017608C10] Jung Y., Bang S., Choi K., Kim E., Kim Y., Kim J., Park J., Koo H., Moon R. T., Song K., et al. (2006). TC1(C8orf4) enhances the Wnt/β-catenin pathway by relieving antagonistic activity of Chibby. *Cancer Res.* 66, 723-728. 10.1158/0008-5472.CAN-05-3124 16424001

[BIO017608C11] Jung Y., Kim M., Soh H., Lee S., Kim J., Park S., Song K. and Lee I. (2014). TC1(C8orf4) regulates hematopoietic stem/progenitor cells and hematopoiesis. *PLoS ONE* 9, e100311 10.1371/journal.pone.0100311 24937306 PMC4061086

[BIO017608C12] Karim R. Z., Tse G. M. K., Putti T. C., Scolyer R. A. and Lee C. S. (2004). The significance of the Wnt pathway in the pathology of human cancers. *Pathology* 36, 120-128. 10.1080/00313020410001671957 15203747

[BIO017608C13] Kim B., Koo H., Yang S., Bang S., Jung Y., Kim Y., Kim J., Park J., Moon R. T., Song K. et al. (2006a). TC1(C8orf4) correlates with Wnt/β-catenin target genes and aggressive biological behavior in gastric cancer. *Clin. Cancer Res.* 12, 3541-3548. 10.1158/1078-0432.CCR-05-2440 16740781

[BIO017608C14] Kim Y., Kim J., Park J., Bang S., Jung Y., Choe J., Song K. and Lee I. (2006b). TC1(C8orf4) is upregulated by IL-1β/TNF-α and enhances proliferation of human follicular dendritic cells. *FEBS Lett.* 580, 3519-3524. 10.1016/j.febslet.2006.05.036 16730711

[BIO017608C15] Kim J., Kim Y., Kim H.-T., Kim D. W., Ha Y., Kim J., Kim C.-H., Lee I. and Song K. (2009). TC1(C8orf4) is a novel endothelial inflammatory regulator enhancing NF-κB activity. *J. Immunol.* 183, 3996-4002. 10.4049/jimmunol.0900956 19684084

[BIO017608C16] Lee I. (2010). 14-3-3 interacts with TC1(C8orf4) to protect the protein from rapid degradation and regulates cancer cell invasiveness. *Cancer Res.* 70, 1248-1248. 10.1158/1538-7445.AM10-1248

[BIO017608C17] Li Y., Han W., Ni T.-T., Lu L., Huang M., Zhang Y., Cao H., Zhang H.-Q., Luo W. and Li H. (2015). Knockdown of microRNA-1323 restores sensitivity to radiation by suppression of PRKDC activity in radiation-resistant lung cancer cells. *Oncol. Rep.* 33, 2821-2828. 10.3892/or.2015.3884 25823795

[BIO017608C18] Liao C., Xiao W., Zhu N., Liu Z., Yang J., Wang Y. and Hong M. (2015). MicroR-545 enhanced radiosensitivity via suppressing Ku70 expression in Lewis lung carcinoma xenograft model. *Cancer Cell Int.* 15, 56 10.1186/s12935-015-0207-z 26041979 PMC4453103

[BIO017608C19] Pérol M., Ciuleanu T.-E., Arrieta O., Prabhash K., Syrigos K. N., Goksel T., Park K., Kowalyszyn R. D., Pikiel J., Lewanski C. R. et al. (2016). Quality of life results from the phase 3 REVEL randomized clinical trial of ramucirumab-plus-docetaxel versus placebo-plus-docetaxel in advanced/metastatic non-small cell lung cancer patients with progression after platinum-based chemotherapy. *Lung Cancer.* 93, 95-103. 10.1016/j.lungcan.2016.01.007 26898621

[BIO017608C20] Ray M. E., Yang Z. Q., Albertson D., Kleer C. G., Washburn J. G., Macoska J. A. and Ethier S. P. (2004). Genomic and expression analysis of the 8p11-12 amplicon in human breast cancer cell lines. *Cancer Res.* 64, 40-47. 10.1158/0008-5472.CAN-03-1022 14729606

[BIO017608C21] Smalley M. J. and Dale T. C. (2001). Wnt signaling and mammary tumorigenesis. *J. Mammary Gland Biol. Neoplasia.* 6, 37-52. 10.1023/A:1009564431268 11467451

[BIO017608C22] Su K., Huang L., Li W., Yan X., Li X., Zhang Z., Jin F., Lei J., Ba G., Liu B. et al. (2013). TC-1 (c8orf4) enhances aggressive biologic behavior in lung cancer through the Wnt/β-catenin pathway. *J. Surg. Res.* 185, 255-263. 10.1016/j.jss.2013.05.075 23880650

[BIO017608C23] Sunde M., McGrath K. C. Y., Young L., Matthews J. M., Chua E. L., Mackay J. P. and Death A. K. (2004). TC-1 is a novel tumorigenic and natively disordered protein associated with thyroid cancer. *Cancer Res.* 64, 2766-2773. 10.1158/0008-5472.CAN-03-2093 15087392

[BIO017608C24] Takemaru K.-I., Yamaguchi S., Lee Y. S., Zhang Y., Carthew R. W. and Moon R. T. (2003). Chibby, a nuclear β-catenin-associated antagonist of the Wnt/Wingless pathway. *Nature* 422, 905-909. 10.1038/nature01570 12712206

[BIO017608C25] Urakami S., Shiina H., Enokida H., Kawakami T., Tokizane T., Ogishima T., Tanaka Y., Li L.-C., Ribeiro-Filho L. A. and Terashima M. (2006). Epigenetic inactivation of Wnt inhibitory factor-1 plays an important role in bladder cancer through aberrant canonical Wnt/β-catenin signaling pathway. *Clin. Cancer Res.* 12, 383-391. 10.1158/1078-0432.CCR-05-1344 16428476

[BIO017608C26] Wang Y.-D., Bian G.-H., Lv X.-Y., Zheng R., Sun H., Zhang Z., Chen Y., Li Q.-W., Xiao Y., Yang Q.-T. et al. (2008). TC1 (C8orf4) is involved in ERK1/2 pathway-regulated G 1-to S-phase transition. *BMB Rep.* 41, 733-738. 10.5483/BMBRep.2008.41.10.733 18959821

[BIO017608C27] Xu H.-T., Liu Y., Liu S.-L., Miao Y., Li Q.-C. and Wang E.-H. (2013). TC-1 (C8orf4) expression is correlated with differentiation in ovarian carcinomas and might distinguish metastatic ovarian from metastatic colorectal carcinomas. *Virchows Arch.* 462, 281-287. 10.1007/s00428-013-1375-7 23377761

[BIO017608C28] Yang Z.-Q., Moffa A. B., Haddad R., Streicher K. L. and Ethier S. P. (2007). Transforming properties of TC-1 in human breast cancer: interaction with FGFR2 and β-catenin signaling pathways. *Int. J. Cancer.* 121, 1265-1273. 10.1002/ijc.22831 17520678

[BIO017608C29] Zhang J., Gao Y., Zhao X., Guan M., Zhang W., Wan J. and Yu B. (2011). Investigation of copy-number variations of C8orf4 in hematological malignancies. *Med. Oncol.* 28, 647-652. 10.1007/s12032-010-9698-6 20878554

[BIO017608C30] Zhang P., Cao H.-Y., Bai L.-L., Li W.-N., Wang Y., Chen S.-Y., Zhang L., Yang L.-H., Xu H.-T. and Wang E.-H. (2015). The high expression of TC1 (C8orf4) was correlated with the expression of β-catenin and cyclin D1 and the progression of squamous cell carcinomas of the tongue. *Tumor Biol.* 36, 7061-7067. 10.1007/s13277-015-3423-1 25869879

